# Icaritin induces lytic cytotoxicity in extranodal NK/T-cell lymphoma

**DOI:** 10.1186/s13046-015-0133-x

**Published:** 2015-02-15

**Authors:** Ting Wu, Songmei Wang, Jinfeng Wu, Zhiguang Lin, Xianxian Sui, Xiaoping Xu, Norio Shimizu, Bobin Chen, Xuanyi Wang

**Affiliations:** Department of Hematology, Huashan Hospital, Shanghai Medical College, Fudan University, 12 Urumqi Middle Road, 200040 Shanghai, China; Laboratory of Molecular Biology, Training Center of Medical Experiments, School of Basic Medical Sciences, Fudan University, Shanghai, China; Department of Integrative Medicine, Huashan Hospital, Shanghai Medical College, Fudan University, Shanghai, China; Department of Physiology & Pathophysiology, School of Basic Medical Sciences, Fudan University, Shanghai, China; Key Laboratory Medical Molecular Virology, MoE/MoH, and the Institutes of Biomedical Sciences, Shanghai Medical College, Fudan University, 131 Dongan Road, 200032 Shanghai, China; Department of Virology, Division of Virology and Immunology, Medical Research Institute, Tokyo Medical and Dental University, Tokyo, Japan

**Keywords:** Icaritin, Extranodal NK/T-cell lymphoma, Apoptosis, EBV, Lytic replication

## Abstract

**Background:**

Extranodal NK/T-cell lymphoma (ENKL) is an aggressive hematological malignancy associated with Epstein–Barr virus (EBV) infection. It is often resistant to conventional chemotherapy and has a poor prognosis. Icaritin, a compound derived from Chinese herbal medicine, Herba Epimedii, has been reported to exert antitumor effects on a variety of cancer cell lines. In the present study, we investigated the cytotoxic effects of Icaritin on the two EBV-positive ENKL cell lines SNK-10 and SNT-8, along with the underlying molecular mechanisms.

**Methods:**

ENKL cell lines SNK-10 and SNT-8 were exposed to different concentrations of Icaritin for the indicated time. Treated cells were analyzed for cell proliferation, cell cycle, and cell apoptosis. Phosphorylation of Stat3 and Akt proteins in signaling pathways and the EBV-encoded LMP1 proteins were measured by Western blot. Expression of EBV genes was assessed by Real-Time PCR.

**Results:**

Our results showed that Icaritin dose-dependently inhibits ENKL cell proliferation and induces apoptosis and cell cycle arrest at G_2_/M phase. Additionally, Icaritin upregulates Bax, downregulates Bcl-2 and pBad, and activates caspase-3 and caspase-9. The anti-proliferative and pro-apoptotic effects of Icaritin are likely mediated by inhibition of Stat3 and Akt pathways through LMP1 downregulation. Importantly, Icaritin induces EBV lytic gene expression in ENKL cells, and the combination of Icaritin and the antiviral drug ganciclovir (GCV) is more effective in inducing ENKL cells apoptosis than Icaritin or GCV alone.

**Conclusions:**

These findings indicate that EBV-targeted approaches may have significant therapeutic potential for ENKL treatment.

## Introduction

Extranodal NK/T-cell lymphoma (ENKL), a specific type of peripheral T-cell lymphoma, is an aggressive malignancy that frequently occurs in the nasal cavity and/or upper aerodigestive tract [1]. ENKL accounts for about 10% of all peripheral T-cell lymphoma and has a higher incidence in Asian and South American populations than in Western countries [2]. Although ENKL is sensitive to radiotherapy, it is inherently resistant to chemotherapy due to expression of p-glycoprotein, and consequently, it has a poorer prognosis than other types of lymphoma [3,4]. Therefore, new therapeutic strategies must be considered in order to improve the clinical outcome of this aggressive malignancy.

Epstein–Barr virus (EBV), one of the most common viruses in humans, is associated with several specific forms of cancer [5]. Specifically, EBV is detected in nearly all ENKL pathology specimens [6], and the geographic localization of ENKL matches the endemic distribution of EBV [3], suggesting that EBV plays an essential role in ENKL etiology. EBV has two distinct life cycles in the human host; a lytic form that produces new infectious virions, and a latent form that allows the virus to persist in a dormant state in the host cell. Based on the expression of EBV-encoded genes, EBV in ENKL specimens is in a latent form exhibiting the “Latency II” gene expression pattern [7].

Recently, new virus-targeted therapeutic approaches have been exploited to treat EBV-associated malignancies [8]. EBV encodes tyrosine kinases that phosphorylate and activate the antiviral drug, ganciclovir (GCV). However, these viral kinases are only expressed during the lytic cycle. Moore and co-workers have reported that induction of EBV lytic reactivation followed by GCV administration produces selective killing of EBV-positive Burkitt’s lymphoma (BL) cells [9]. Recently, an increasing number of reports have shown that activating the EBV latent-lytic switch could be therapeutically beneficial for EBV-associated tumors such as BL, nasopharyngeal carcinoma (NPC), and gastric carcinoma (GC) [10-12]. Arginine butyrate, a general histone deacetylase inhibitor (HDACi), acts as an inducer of EBV lytic-phase gene expression [13]. Arginine butyrate in combination with GCV was used to treat refractory lymphomas in a phase I/II clinical trial and achieved significant therapeutic results [14]. However to our knowledge, no agent has been reported to effectively induce EBV lytic re-activation in EBV-positive ENKL.

**Figure 1:**
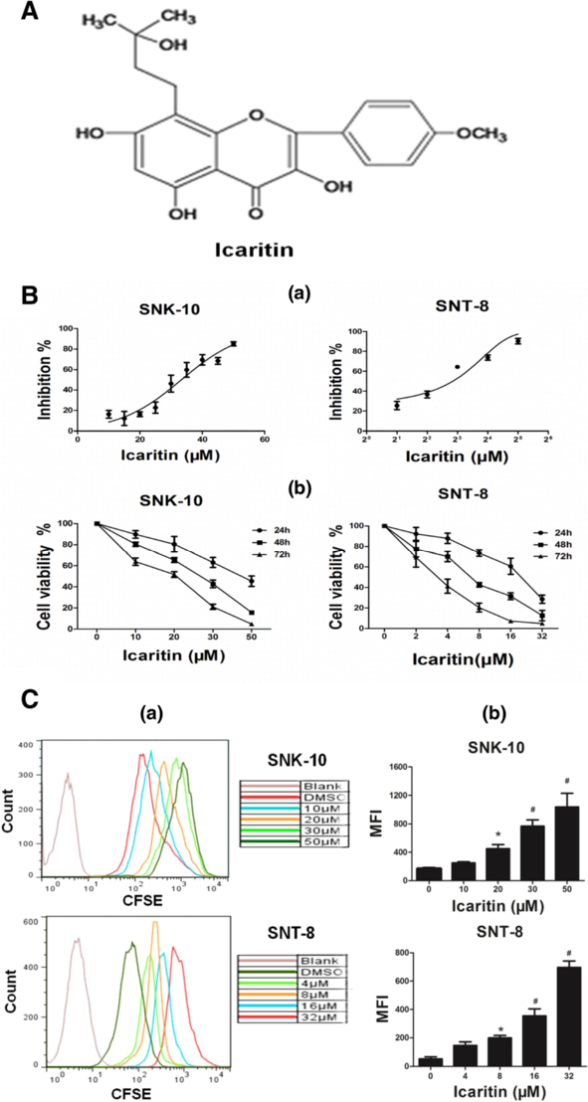
**Icaritin exhibits cytotoxicity on ENKL cells. A**. The chemical structure of Icaritin. **B**. Effects of Icaritin on SNK-10 and SNT-8 cell viability by the CCK-8 assay. **(a)** IC_50_ curves after 72 h treatment (n = 3); **(b)** Time- and dose–response curves (n = 3). **C**. Effects of Icaritin on SNK-10 and SNT-8 cell proliferation. Cell proliferation was determined by the CFDA-SE assay after 48 h treatment. **(a)** CFDA-SE flow cytometry histograms; **(b)** The mean cellular florescence intensity (MFI) (n = 3). *p < 0.05, ^#^p < 0.01 vs control.

Icariin is a major active ingredient of the traditional Chinese herbal medicine Epimedium. Icaritin (Figure 1A), an intestinal metabolite of Icariin has been shown to exhibit a variety of beneficial biological activities including neuroprotective [15] and anti-inflammatory properties [16,17]. Recent studies have shown that Icaritin also exhibits anti-cancer properties. Icaritin inhibits growth and induces apoptosis in breast and endometrial cancer cells through sustained ERK activation [18,19]. In hematological malignancy, Icaritin strongly inhibits growth of primary chronic myeloid leukemia (CML) cells *in vitro* and *in vivo* by regulating the MAPK/ERK/JNK and JAK2/STAT3/AKT pathways [20]. Icaritin also inhibits growth and triggers apoptosis of acute myeloid leukemia (AML) cells via downregulation of the MAPK/ERK and PI3K/AKT signals [21]. However, it is not known whether Icaritin possesses anti-ENKL activity.

In the present study, we found that Icaritin inhibited growth and induced apoptosis and cell cycle arrest at G2/M phase in the ENKL cell lines SNK-10 and SNT-8. We also demonstrated that Icaritin is an effective inducer of EBV lytic-phase gene expression in ENKL cell lines, and Icaritin in combination with GCV induced apoptosis in EBV-positive ENKL cells more effectively. These findings suggest the potential clinical application of Icaritin as a novel therapy against EBV-positive ENKL.

## Materials and methods

### Reagents

Icaritin was purchased from Shanghai Ronghe (Shanghai, China). A stock solution was prepared by dissolving Icaritin in DMSO (Sigma, Louis, MO, USA) and stored at −20°C. The final concentration of DMSO in the treatment medium was controlled below 0.1%. GCV was purchased from Hubei Ke Yi Pharmaceutic Co., Ltd (Hubei, China). Antibodies against caspase-9, caspase-3, Bax, Stat3, and p-Stat3 (pY705) were purchased from Epitomics (Burlingame, CA, USA). Antibodies against Bcl-2 and pAkt (Ser473) were from Cell Signaling Technology (Boston, MA, USA). Antibodies against Bad (F130) and pBad (S136) were from Bioworld Technology, Inc. (Louis Park, MN, USA). LMP1 antibody and HRP-conjugated goat anti-mouse/rabbit secondary antibody were from Abcam (Cambridge, MA, USA). EBV Zta antibody was from Santa Cruz biotechnology (Dallas, TX, USA). β-Tubulin antibody was from Beijing CoWin Bioscience Co., Ltd (Beijing, China).

### Cells and cell culture

The ENKL cell lines, SNK-10 and SNT-8, were provided by Dr. Norio Shimizu at Tokyo Medical and Dental University. SNK-10 was established from the peripheral blood of an ENKL patient with chronic active EBV infection [22]. SNT-8 was derived from primary lesions of a Japanese patient with EBV-positive ENKL [23]. SNK-10 and SNT-8 cells were cultured in RPMI-1640 (Hyclone) media supplemented with 10% heat-inactivated human plasma, 1% penicillin-streptomycin, and 700 U/ml of recombinant human interleukin-2 (IL-2) (Peprotech, Rochy Hill, NJ, USA).

### Cell viability and proliferation assays

Cell viability was measured using the CCK-8 assay (Beyotime, Shanghai, China) following manufacturer’s instructions. The percent of viable cells was calculated using the formula: ratio (%) = [OD (Treatment) – OD (Blank)]/[OD (Control) – OD(Blank)] × 100. Each experiment was carried out in six replicates and results were calculated over three independent experiments.

Cell proliferation was determined using the CFDA-SE Cell Proliferation Assay (Beyotime). Cells were stained with carboxyfluorescein diacetate succinimidyl ester (CFDA-SE) according to manufacturer’s instructions and cultured in six-well plates with various concentrations of Icaritin for 48 h. CFDA-SE dilution was analyzed by flow cytometry on a FACSCalibur (BD Biosciences, CA, USA) and data were analyzed using the FlowJo software (Treestar, Ashland, OR, USA).

### Cell cycle analysis

Cells were incubated with vehicle (0.1% DMSO) or different concentrations of Icaritin for 48 h, harvested, and fixed by incubation in 70% ethanol (500 μl) at 4°C overnight. Cells were then collected by centrifugation at 900Xg for 5 min and washed with PBS. Subsequently, cells were incubated with 100 μl RNaseA (KeyGEN, Nanjing, China) at 37°C for 30 min, and then with 400 μl propidium iodide (PI) at 4°C for 30 min in the dark. DNA content was analyzed on a FACSCalibur flow cytometer and data were analyzed using the Modfit LT 3.2 software (Verity Software House, ME, USA).

### Cell apoptosis analysis

Cells were seeded in six-well plates and incubated with various concentrations of Icaritin for 48 h. Cell apoptosis was determined using the annexin V-FITC apoptosis kit (BestBio, Shanghai, China) following manufacturer’s instructions. Cells were analyzed on a FACScan flow cytometer (BD Biosciences) and data were interpreted using the Flowjo software (Treestar).

### Analysis of nuclear morphology

Cells were incubated with Icaritin or vehicle for 48 h, harvested, and fixed by incubating in stationary liquid for 10 min at room temperature. After being washed twice with PBS, cells were stained with Hoechst-33258 (Beyotime) and examined by confocal florescence microscopy (Nikon, Tokyo, Japan). The apoptotic cells exhibited nuclear shrinkage, and condensed, fragmented chromatin structure as well.

### Real-Time PCR

Total RNA was extracted using Trizol Reagent (Invitrogen, Carlsbad, CA, USA) following manufacturer’s instructions. cDNA was synthesized using PrimeScript RT Master Mix (Perfect Real Time) (Takara, Tokyo, Japan) and subjected to Real-Time PCR using a SYBR Premix Ex Taq (Tli RNaseH Plus) (Takara) on a 7500 Real-Time PCR System (ABI, Grand Island, NY, USA). All reactions were performed in duplicate. Primers used for Real-Time PCR are shown in Table 1.

**Table 1:** **Primers used for Real-Time PCR**

**Target gene**	**Sequence (5′–3′)**
GAPDH-F	AGAAGGCTGGGGCTCATTTG
GAPDH-R	AGGGGCCATCCACAGTCTTC
LMP1-F	CCCTTTGTATACTCCTACTGATGATCAC
LMP1-R	ACCCGAAGATGAACAGCACAAT
BZLF1-F	CATGTTTCAACCGCTCCGACTGG
BZLF1-R	GCGCAGCCTGTCATTTTCAGATG
BMRF1-F	ACCTGCCGTTGGATCTTAGTG
BMRF1-R	GGCGTTGTTGGAGTCCTGTG
BRLF1-F	GAAGCCCGGTGCCCAAAG
BRLF1-R	GTGTCACTGTTGCCCGAGTC
EBNA1-F	CGTTTGGGAGAGCTGATTCT
EBNA1-R	CCCCTCGTCAGACATGATTC

### Western blot analysis

Cells following treatment were lysed with RIPA (Beyotime) in the presence of the protease inhibitor PMSF (Beyotime). Protein concentration was determined using the BCA protein assay kit (Beyotime). Protein samples (30 μg each) were separated by SDS-PAGE and transferred to PVDF membranes (Immobilon-P membrane; Millipore, USA). After incubation in 5% fat-free milk at room temperature for 2 h to block non-specific binding, membranes were incubated with primary antibodies in TBS-Tween (TBST) (0.05% Tween-20 in TBS) at 4°C overnight. Membranes were then washed three times with TBST and incubated with secondary antibody for 1 h at room temperature. Membranes were again washed three times with TBST and visualized by enhanced chemiluminescence using Supersignal West Pico Trial Kit (Thermo Scientific, Massachusetts, USA).

### Statistical analysis

Results are presented as means ± standard deviation (SD). The dose–response analyses were performed by one-way ANOVA with Dunnett’s-t test. Cell apoptosis rate was entered into 2 × 2 factorial design with Icaritin and GCV as between-subject factors. All statistical analyses were performed using the software Stata 12.0 for windows. Differences with p < 0.05 were considered statistically significant.

## Results

### Icaritin exhibits cytotoxicity towards ENKL cells *in vitro*

We first investigated the effects of Icaritin on SNK-10 and SNT-8 cell viability using the CCK-8 assay. We found that Icaritin decreased SNK-10 and SNT-8 cell viability in a time and dose-dependent manner (Figure 1B-b), with an IC_50_ of 28 μM toward SNK-10 cells and 6.5 μM toward SNT-8 cells after 72 h treatment (Figure 1B-a). We further examined the effects of Icaritin on ENKL cell proliferation using the CFDA-SE cell proliferation assay. SNK-10 and SNT-8 cells were stained with CFDA-SE and incubated with Icaritin for 48 h. Cell proliferation was determined by analysis of CFDA-SE dilution on a flow cytometer. CFDA-SE histograms showed that Icaritin dose-dependently increased cellular florescence intensity of SNK-10 and SNT-8 cells (Figure 1C-a), indicating that Icaritin inhibited SNK-10 and SNT-8 cell division. Specifically, Icaritin at 20, 30, and 50 μM significantly increased the mean florescence intensity (MFI) of SNK-10 cells to 451.7 (p < 0.05), 767.3 (P < 0.01), and 1039.3 (P < 0.01), respectively, compared with 164.3 of untreated cells (Figure 1C-b). Similar to results from the cell viability assay, SNT-8 cells were more sensitive to Icaritin-induced growth inhibition compared with SNK-10 (Figure 1C-b). Collectively, these results indicated that Icaritin exhibits cytotoxicity towards ENKL cells *in vitro*.

### Icaritin induces cell cycle arrest at G_2_/M phase in ENKL cells

**Figure 2:**
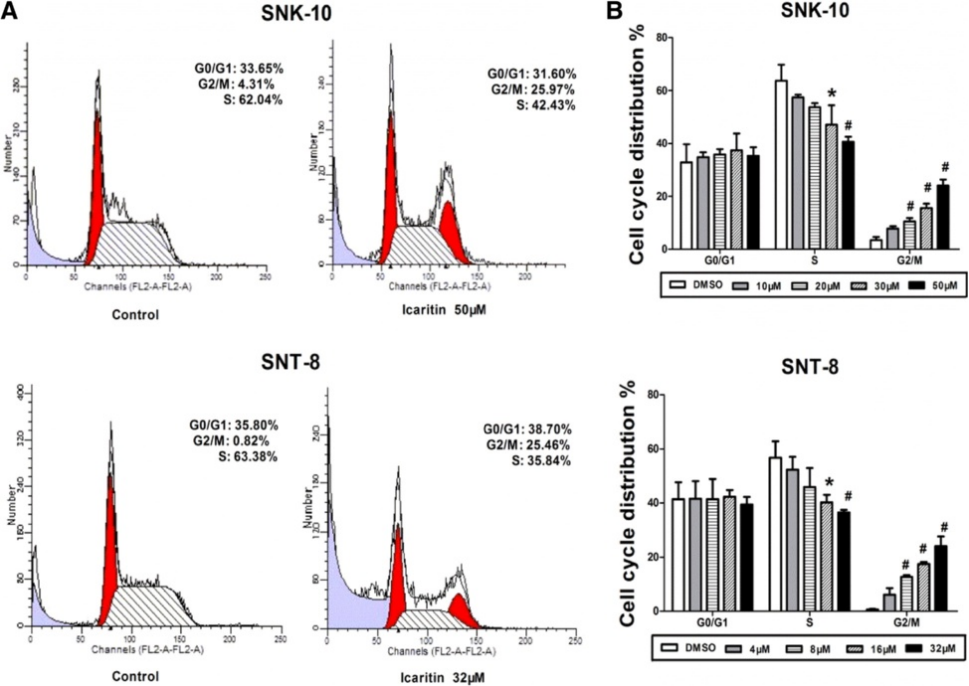
**Icaritin induces cell cycle arrest at G**_**2**_ **/M phase in ENKL cells.** Cell cycle distribution of SNK-10 and SNT-8 cells was analyzed by flow cytometry with PI staining after 48 h treatment. **A**. Representative flow cytometry histograms. **B**. Cell distribution at G_0_/G_1_, S, and G_2_/M phases of the cell cycle (n = 3). *p < 0.05, ^#^p < 0.01 vs control.

We evaluated the effects of Icaritin on cell cycle distribution of SNK-10 and SNT-8 by flow cytometry with PI staining. Our data showed that Icaritin treatment significantly increased the proportion of cells at G_2_/M phase, which was accompanied by a decreased proportion at S phase (Figure 2). Specifically, untreated SNK-10 and SNT-8 cells had only 3.5% and 0.6% cells, respectively, at G_2_/M phase. Treatment of SNK-10 and SNT-8 cells with 50 μM and 32 μM Icaritin significantly increased the proportion of cells at G_2_/M phase to 22.4% and 24.1%, respectively. Moreover, while untreated SNK-10 and SNT-8 cells had 63.7% and 56.7% cells at S phase, respectively, treatment with 50 μM and 32 μM Icaritin significantly reduced the proportion of cells at S phase to 42.2% and 36.5%, respectively (Figure 2B). These results suggested that Icaritin inhibits ENKL cell proliferation through inducing cell cycle arrest at G_2_/M phase.

### Icaritin induces apoptosis in ENKL cells

**Figure 3:**
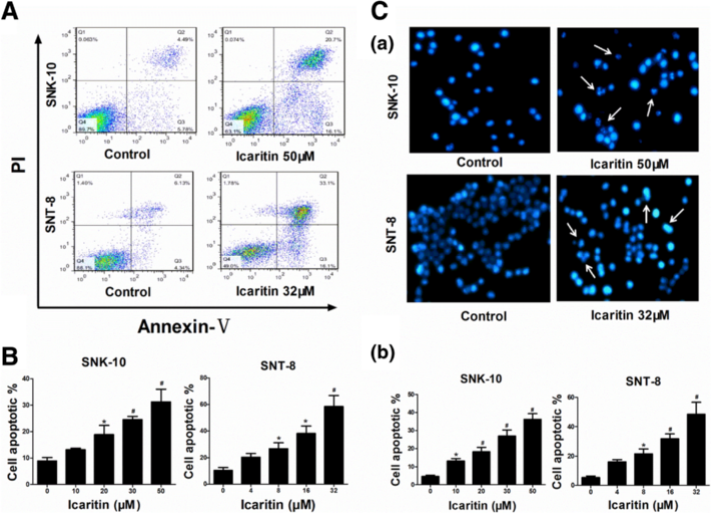
**Icaritin induces ENKL cell apoptosis.** Cell apoptosis of SNK-10 and SNT-8 was analyzed by flow cytometry and florescence microscopy, respectively, after 48 h treatment with Icaritin. **A**. Representative histograms of annexin V-FITC/PI double-staining flow cytometry. **B**. Percent apoptosis (including early and late apoptotic cells) determined by flow cytometry (n = 3). **C**. Cell apoptosis assessed by florescence microscopy. Cells were stained with Hoechst 33258. **(a)** Cell and nuclear morphology by florescence microscopy (magnification 400×). The arrows indicate the cells undergoing apoptosis; **(b)** Percent apoptosis determined by morphology-based cell sorting (n = 3). At least 1,000 cells from five randomly selected fields were counted for each data point. *p < 0.05, ^#^p < 0.01 vs control.

Many chemotherapeutic agents promote cancer cell apoptosis. We investigated the effects of Icaritin on ENKL cell apoptosis using annexin V/PI dual staining flow cytometry. Our data showed that Icaritin induced apoptosis in both SNK-10 and SNT-8 cells (Figure 3A and B). As shown in the flow cytometry histograms (Figure 3A), Icaritin dose-dependently increased the population of early and late apoptotic cells. While untreated SNK-10 and SNT-8 cells each had about 10% apoptosis, SNK-10 cells treated with 50 μM Icaritin showed 32% apoptosis, and SNT-8 cells treated with 32 μM displayed 58% apoptosis (Figure 3B). These results indicated that Icaritin induces apoptosis in ENKL cells.

We also studied the effects of Icaritin on ENKL cell morphology. The blue fluorescent Hoechst dyes are cell permeable nucleic acid stains that are very sensitive to DNA conformation and chromatin state in cells. Consequently, they can detect gradations of nuclear damage. After 48 h treatment with Icaritin, SNK-10 and SNT-8 cells were stained with Hoechst 33258 and examined by florescence microscopy. As shown in Figure 3C-a, SNK-10 and SNT-8 cells treated with Icaritin exhibited apoptotic morphological characteristics such as cell shrinkage and chromatin condensation and fragmentation, while these morphological features were not evident in untreated cells. In addition, the percent of apoptotic cells determined by morphology-based cell sorting (Figure 3C-b) was similar to that obtained by annexin V/PI dual staining flow cytometry (Figure 3B).

### Icaritin induces caspase activation in ENKL cells

**Figure 4:**
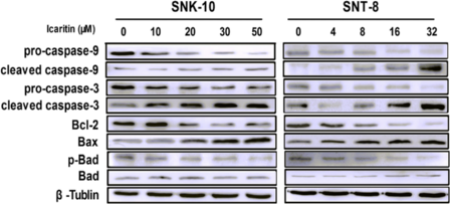
**Effects of Icaritin on caspases and Bcl-2 family proteins in ENKL cells.** SNK-10 and SNT-8 cells were subjected to western blot analysis after 48 h treatment with Icaritin. β-tubulin was used as an internal control. Representative blots are shown.

To investigate the mechanisms underlying Icaritin’s pro-apoptotic effects in ENKL cells, we determined the levels of caspases and the Bcl-2 family proteins by western blot analysis. Our results showed that Icaritin dose-dependently increased levels of cleaved caspase-9 and caspase-3 in SNK-10 and SNT-8 cells (Figure 4). We also found that Icaritin down-regulated Bcl-2 and p-Bad and up-regulated Bax in both cell lines while having no significant effects on Bad expression (Figure 4). Collectively, these results suggested that Icaritin induces ENKL cell apoptosis through activating the mitochondria-mediated caspase pathway.

### Icaritin inhibits Stat3 and Akt signaling in ENKL cells

**Figure 5:**
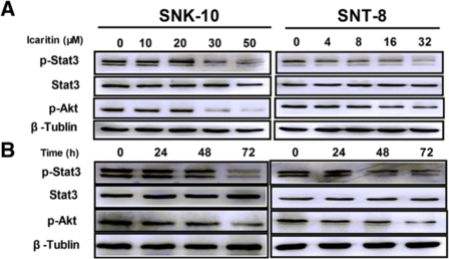
**Effects of Icaritin on Stat3 and Akt signaling pathways in ENKL cells.** Protein levels in SNK-10 and SNT-8 cells were determined by western blot analysis after treatment. **A**. Cells were treated with Icaritin at indicated concentrations for 48 h. **B**. Cell were treated with 30 μM (SNK-10) or 16 μM (SNT-8) Icaritin for indicated time. β-tubulin was used as an internal control. Representative blots are shown.

To further investigate the mechanisms of action of Icaritin, we analyzed the effects of Icaritin on the proliferative and survival signals Stat3 and Akt in ENKL cells. After 48 h treatment with Icaritin, SNK-10 and SNT-8 cells were lysed and subjected to western blot analysis. As shown in Figure 5, Icaritin reduced p-Stat3 and p-Akt levels in a dose- and time-dependent manner in both cell lines without affecting the level of total Stat3. These results suggested that Icaritin inhibits growth and induces apoptosis in ENKL cells likely through regulation of the Stat3 and Akt signaling pathways.

### Icaritin reduces LMP1 mRNA and protein expression in ENKL cells

**Figure 6:**
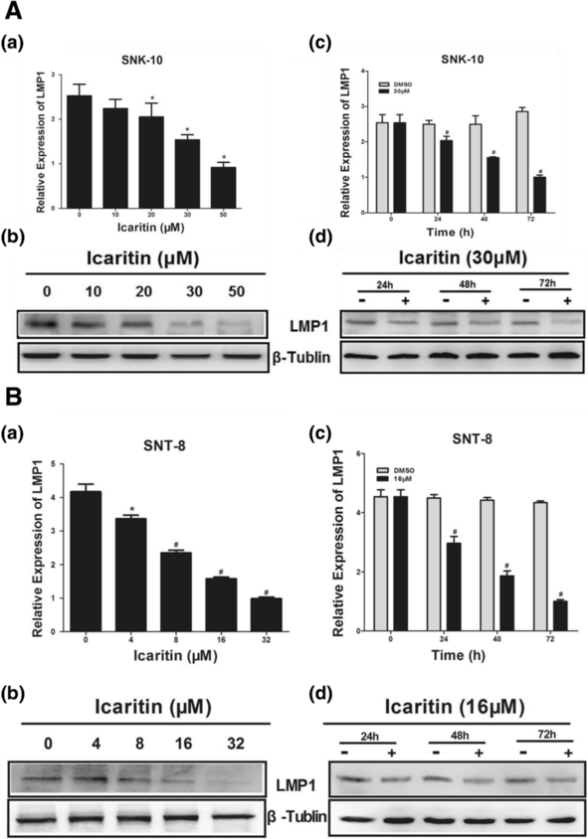
**Effects of Icaritin on LMP1 expression in ENKL cells. A-a** **(B-a)**. SNK-10 cells (SNT-8 cells) were treated with Icaritin at indicated concentrations for 48 h and LMP1 mRNA level was determined by Real-Time PCR. **A-b** **(B-b)**. SNK-10 cells (SNT-8 cells) were treated with Icaritin at indicated concentrations for 48 h and LMP1 protein level was determined by western blot analysis. **A-c** **(B-c)**. SNK-10 cells (SNT-8 cells) were treated with 30 μM (16 μM) Icaritin for indicated time and LMP1 mRNA level was determined by Real-Time PCR. **A-d** **(B-d)**. SNK-10 cells (SNT-8 cells) were treated with 30 μM (16 μM) Icaritin for indicated time and LMP1 protein level was determined by western blot analysis. *p < 0.05, ^#^p < 0.01.

EBV-encoded LMP1 protein activates proliferative and survival signals including NF-κB, MAPK, JAK/STAT, and AKT to regulate the proliferation, immortalization, and invasion of infected lymphoma cells [24-26]. We examined the effects of Icaritin on LMP1 mRNA and protein expression in ENKL cells by Real-Time PCR and western blot analysis, respectively. We found that after 48 h treatment, Icaritin at 30 μM and 50 μM significantly reduced LMP1 mRNA and protein expression in SNK-10 cells (Figure 6A-a and A-b). We also found that Icaritin at 30 μM inhibited LMP1 mRNA and protein expression in a time-dependent manner, with significant reduction observed after 24, 48, and 72 h incubation (Figure 6A-c and A-d). As the Figure 6B shown, Icaritin reduced LMP1 mRNA and protein expression in a dose- and time-dependent manner in SNT-8 cells. These data suggested that the inhibitory effects of Icaritin on Stat3 and Akt signaling in ENKL cells might be mediated by downregulation of LMP1 expression.

### Icaritin induces EBV lytic-phase gene expression and sensitizes ENKL cells to GCV treatment

**Figure 7:**
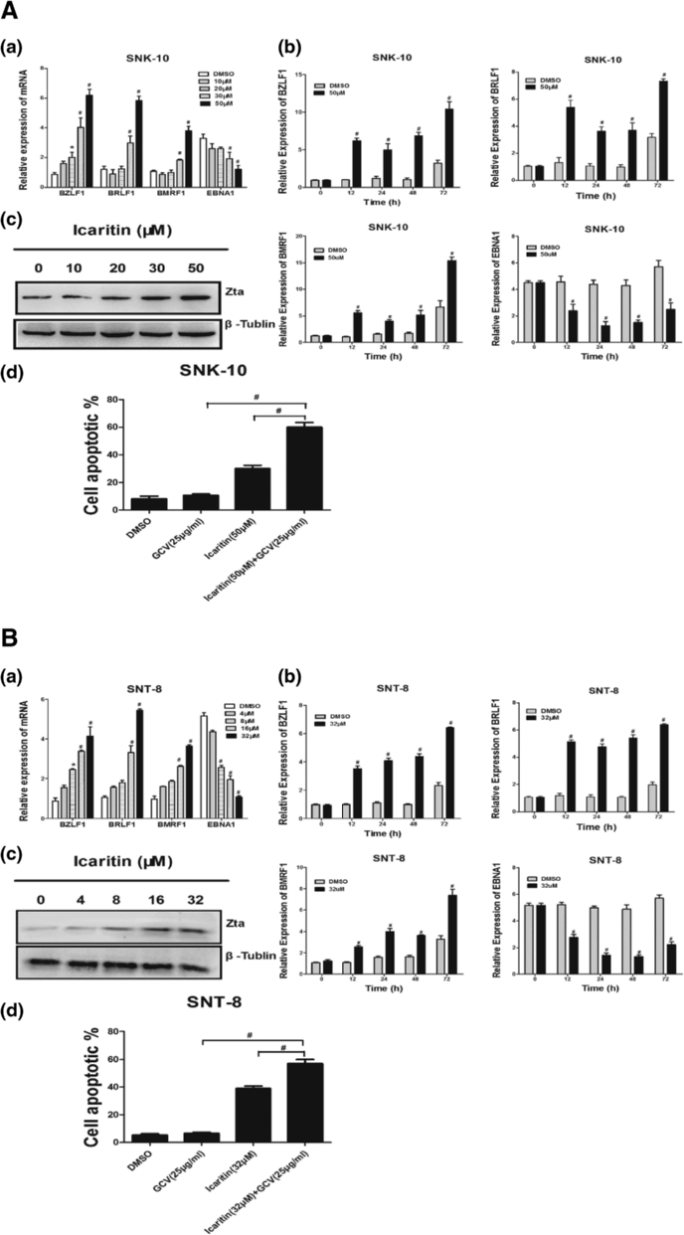
**Icaritin induces EBV lytic-phase gene expression and sensitizes ENKL cells to GCV treatment. A-a** **(B-a)**. ENKL cells were treated with Icaritin at indicated concentrations for 48 h and the mRNA levels of EBV-encoded genes (BZLF1, BRLF1, BMRF1, and EBNA1) were determined by Real-Time PCR (n = 3). *p < 0.05, ^#^p < 0.01 vs control. **A-b** **(B-b)**. SNK-10 cells (SNT-8 cells) were treated with 50 μM (32 μM) Icaritin or DMSO for indicated time and the mRNA levels of EBV-encoded genes (BZLF1, BRLF1, BMRF1, and EBNA1) were determined by Real-Time PCR (n =3). *p < 0.05, ^#^p < 0.01 vs control. **A-c (B-c)**. SNK-10 cells (SNT-8 cells) were treated with Icaritin at indicated concentrations for 48 h and the protein level of the IE protein Zta was determined by western blot analysis. **A-d (B-d)**. Icaritin sensitizes ENKL cells to GCV treatment. SNK-10 and SNT-8 cells were treated respectively with Icaritin, GCV, or both for 48 h and the cell apoptosis was determined by flow cytometry with annexin V-FITC/PI double-staining (n = 3). ^#^p < 0.01.

Finally we studied the effects of Icaritin on EBV latent-lytic switch in ENKL cells by analyzing the mRNA expression of three lytic-phase genes and one latent-phase gene. The three lytic-phase genes included the two EBV immediate early (IE) genes BZLF1 and BRLF1, and the early gene BMRF1. The latent-phase gene EBNA1 is expressed in EBV at all latency stages. After 48 h treatment, Icaritin significantly induced the mRNA expression of the three lytic-phase genes (BZLF1, BRLF1, and BMRF1), and inhibited the mRNA expression of the latent-phase gene (EBNA1) in a dose-dependent manner (Figure 7A-a and B-a). In addition, a time-course study showed that 50 μM Icaritin for SNK-10 and 32 μM for SNT-8 significantly upregulated the three lytic genes and downregulated the latent gene after 12, 24, 48, and 72 h incubation (Figure 7A-b and B-b). Since expression of EBV IE protein Zta (also referred to as BZLF1, ZEBRA, Z or EB1) is sufficient to activate the entire cascade of lytic EBV infection, we examined the expression of Zta (BZLF1) protein after Icaritin treatment. Similar to the effects observed on the mRNA expression, Icaritin dose-dependently increased the Zta protein expression in both cell lines (Figure 7A-c and B-c). Taken together, these results suggested that Icaritin promotes lytic EBV infection in EBV-positive ENKL.

Since EBV-encoded tyrosine kinases expressed during the lytic cycle phosphorylate and convert the antiviral prodrug GCV into its active form, we speculated that Icaritin might sensitize ENKL cells to GCV treatment through induction of lytic EBV infection. Indeed, we found that treatment of ENKL cells simultaneously with Icaritin (50 μM for SNK-10 and 32 μM for SNT-8) and GCV (25 μg/ml) for 48 h triggered significantly more severe apoptosis than with either Icaritin or GCV alone (Figure 7A-d and B-d).

## Discussion

Extranodal natural killer/T-cell lymphoma (ENKL) is a highly aggressive hematological malignancy associated with Epstein–Barr virus (EBV) infection. It is often resistant to conventional chemotherapy and new therapeutic approaches need to be considered to achieve better clinical outcome [27]. Icaritin is a compound derived from Chinese herbal medicine Herba Epimedii, which is traditionally used in East Asian countries to improve bone health [28] and treat menopausal symptoms [29]. Recently, Icaritin has been reported to exert antitumor effects on various cancer cell lines. In this study, we investigated the cytotoxic effects of Icaritin on the EBV-positive ENKL cell lines SNK-10 and SNT-8.

We found that Icaritin effectively inhibited cell viability of the two ENKL cell lines with IC_50_ values of 28 μM and 6.5 μM for SNK-10 and SNT-8, respectively, after 72 h treatment. The inhibitory effects of Icaritin on the two ENKL cell lines were also observed on proliferation index in a CFDA-SE proliferation assay. Furthermore, our results from flow cytometry analysis demonstrated that Icaritin induced cell apoptosis and G_2_/M arrest in SNK-10 and SNT-8, similar to previously reported effects of Icaritin on breast cancer cells [18,30].

Icaritin has been reported to inhibit growth and induce apoptosis in cancer cells through several mechanisms that include: 1) upregulation of pro-apoptotic proteins (i.e. Bax and Bak) and downregulation of anti-apoptotic proteins (i.e. Bcl-2) [19,31]; and 2) inhibition of PI3K/AKT, Jak/Stat, and MAPK/ERK signaling pathways [20,21]. In this study, we found that Icaritin upregulated pro-apoptotic protein Bax and downregulated anti-apoptotic protein Bcl-2 and p-Bad, along with cleaved caspase-3 and cleaved caspase-9. These results suggested that mitochondria-mediated caspase cascade plays a major role in Icaritin-induced ENKL cell apoptosis.

Proliferative and survival Stat3 and Akt pathways are activated in ENKL cell lines as well as in the neoplastic cells of most ENKL primary tumors [32,33]. Inhibition of activated Stat3 in the ENKL cell line MEC04 induced cell death. Additionally, treatment of MEC04 with the JAK inhibitor AG490 led to inhibited cell growth along with decreased level of p-Stat3 [34]. Phosphorylated Akt is detected in ENKL [35]. Treatment of NK-92 with the PI3K inhibitor LY294002 resulted in a reduction of p-Akt and cell apoptosis [36]. Taken together, these studies highlight the importance of JAK/STAT and PI3K/AKT pathways in ENKL survival, and thus targeting Stat and Akt might be an effective therapeutic approach for ENKL. In this present study, we found that Icaritin reduced levels of p-Stat3 and p-Akt in SNK-10 and SNT-8, suggesting that the inhibitory effects of Icaritin on ENKL cells are mediated by inhibition of the Jak/Stat3 and Akt signaling pathways.

EBV-encoded LMP1 protein activates Stat3 and Akt in EBV-associated malignancies including B cell lymphoma [37], NPC [38], and ENKL [39]. Interestingly, we found that Icaritin downregulated the mRNA and protein levels of LMP1 in ENKL cells, suggesting that the inhibitory effects of Icaritin on Stat3 and Akt might be mediated by reduction of LMP1 expression.

Recent studies have shown that specific chemotherapeutic agents induce EBV lytic replication in EBV-positive cancer cells [12,40], and by doing so, sensitize cancer cells to nucleoside antiviral agents [13]. Reactivation of EBV lytic cycle begins with the expression of the EBV immediate early (IE) viral gene BZLF1, which encodes the transcriptional activator Zta [41,42]. Zta subsequently activates the other IE gene BRLF1 [43]. BZLF1 and BRLF1 together activate the early lytic gene BMRF1, which encode viral proteins responsible for replication [44]. In this study, we found that Icaritin at 50 μM and 32 μM for SNK-10 and SNT-8, respectively, significantly induced expression of the EBV lytic genes BZLF1, BRLF1, and BMRF1 after 48 h incubation. In addition, our time-course study showed that Icaritin significantly increased EBV lytic gene expression after 12 h – 72 h incubation in both cell lines. Sivachandran et al. [45] reported that depletion of EBV viral gene EBNA1 in latently infected cells positively contributes to spontaneous EBV re-activation, showing that EBNA1 has a role in suppressing re-activation. In this study, we found that Icaritin significantly reduced the expression of EBNA1 in SNK-10 and SNT-8. Collectively, these results suggested that Icaritin activates EBV lytic replication in ENKL cells.

During the EBV lytic cycle, viral kinases are expressed that phosphorylate the antiviral pro-drug GCV into its active cytotoxic form [46]. Phosphorylated GCV, a nucleoside analogue, inhibits not only the viral DNA polymerase but also the host cell DNA polymerase, and thereby kills both EBV and EBV-infected cancer cells [42]. Therefore, agents inducing lytic EBV infection sensitize tumor cells to GCV treatment [8]. In the present study, we showed that the combination of GCV and Icaritin was much more effective in inducing ENKL cell apoptosis than either agent alone. To our knowledge, this is the first report that Icaritin activates lytic EBV infection and sensitizes ENKL to GCV treatment.

In summary, we demonstrate that Icaritin inhibits cell proliferation and induces cell apoptosis and cell cycle arrest at G_2_/M phase in ENKL cells. These effects are possibly mediated by inhibition of Jak/Stat3 and PI3K/AKT pathways via downregulation of LMP1. In addition, Icaritin activates lytic EBV infection and sensitizes ENKL cells to antiviral treatment by GCV. Our results provide rationale that EBV-targeted agents have therapeutic potential for ENKL treatment.

## Abbreviations

ENKL: Extranodal natural killer/T-cell lymphoma
EBV: Epstein–Barr virus
GCV: Ganciclovir
LMP1: Latent membrane protein 1
IE: Immediate early
CFDA-SE: Carboxyfluorescein diacetate succinimidyl ester
MFI: Mean florescence intensity
